# Pathogenic Potential of Opportunistic Gram-Negative Bacteria Isolated from the Cloacal Microbiota of Free-Living Reptile Hosts Originating from Bulgaria

**DOI:** 10.3390/life14050566

**Published:** 2024-04-27

**Authors:** Irina Lazarkevich, Stephan Engibarov, Simona Mitova, Steliyana Popova, Emiliya Vacheva, Nikola Stanchev, Rumyana Eneva, Yana Gocheva, Iva Lalovska, Tsvetelina Paunova-Krasteva, Yana Ilieva, Hristo Najdenski

**Affiliations:** 1 The Stephan Angeloff Institute of Microbiology, Bulgarian Academy of Sciences, 26 Georgi Bonchev Str., 1113 Sofia, Bulgaria; stefan_engibarov@abv.bg (S.E.); simona.mitova9@abv.bg (S.M.); rum_eneva@abv.bg (R.E.); yana2712@gmail.com (Y.G.); pauny@abv.bg (T.P.-K.); illievayana@gmail.com (Y.I.); hnajdenski@gmail.com (H.N.); 2 Faculty of Biology, Sofia University “St. Kliment Ohridski”, 8 Dragan Tsankov Blvd., 1164 Sofia, Bulgaria; steliyanski@gmail.com (S.P.); nickolastanchev@abv.bg (N.S.); 3 Institute of Biodiversity and Ecosystem Research, Bulgarian Academy of Sciences, 1 Tsar Osvoboditel Blvd., 1000 Sofia, Bulgaria; emilia.vacheva@gmail.com; 4 Tortoise Rescue, Rehabilitation and Breeding Center, Gea Chelonia Foundation, 10 Shipka Street, Banya Village, 8239 Burgas, Bulgaria; office@geachelonia.org

**Keywords:** cloacal microbiota, reptiles, lizards, tortoises, antibiotic resistance, biofilm formation, exoenzyme–virulence factors

## Abstract

Reptiles are known to be asymptomatic carriers of various zoonotic pathogens. A number of Gram-negative opportunistic commensals are causative agents of bacterial infections in immunocompromised or stressed hosts and are disseminated by reptiles, whose epidemiological role should not be neglected. Since most studies have focused on exotic species, in captivity or as pet animals, the role of wild populations as a potential source of pathogens still remains understudied. In the present study, we isolated a variety of Gram-negative bacteria from the cloacal microbiota of free-living lizard and tortoise hosts (Reptilia: Sauria and Testudines) from the Bulgarian herpetofauna. We evaluated their pathogenic potential according to their antibiotic susceptibility patterns, biofilm-forming capacity, and extracellular production of some enzymes considered to play roles as virulence factors. To our knowledge, the phenotypic manifestation of virulence factors/enzymatic activity and biofilm formation in wild reptile microbiota has not yet been widely investigated. All isolates were found to be capable of forming biofilms to some extent and 29.6% of them could be categorized as strong producers. Two strains proved to be excellent producers. The majority of the isolated strains showed extracellular production of at least one exoenzyme. The most pronounced pathogenicity could be attributed to the newly isolated *Pseudomonas aeruginosa* strain due to its multiresistance, excellent biofilm formation, and expression of exoenzymes.

## 1. Introduction

Reptiles are known to be asymptomatic carriers of various pathogens, such as viruses, protozoa, fungi, parasites, and, most often, bacteria, that in certain cases could be transmitted to other animals and humans [1,2]. The most common bacterial zoonoses whose vectors appear to be reptiles are salmonellosis [3,4,5], leptospirosis [6], and infections by *Mycobacterium*, *Chlamydia*, *Aeromonas*, and *Pseudomonas* [1,7,8,9,10]. However, most studies have focused on exotic species, namely animals kept in captivity or as pets [5,11,12]. Reptiles are popular as pets in many countries, e.g., across Europe, where the number of captive reptiles has been estimated at over 9 million in recent years [13]. Also, many bacterial diseases, common in reptiles, involve Gram-negative opportunistic commensals that could infect malnourished, poorly maintained, or immunosuppressed hosts living under stressful conditions [11,14]. A number of isolates, identified as the genera *Pseudomonas*, *Serratia*, *Salmonella*, and *Klebsiella* or the species *Citrobacter freundii*, *Morganella morganii*, *Escherichia coli*, etc., and found often in combination, are among the recognized bacterial causative agents that have been recovered from reptile abscesses [14]. However, the health status of wild populations remains largely unclear and their role as a potential source of pathogens is still understudied. In general, investigations on the cloacal microbiota of free-living reptile hosts are few in Europe [15,16,17,18,19], but all of them indicate that their epidemiological potential should not be neglected. Research on isolated Gram-negative bacteria as opportunistic human pathogens has emphasized their capability of causing severe infections, especially in some risk groups. The moderate to high prevalence of *Salmonella* in fecal samples from snakes, turtles, and lizards suggests a possible hazard [20,21,22]. Besides direct human–animal contact, in some cases, the transfer of infection is possible indirectly through a contaminated environment [13,16]. Therefore, following the global “One Health” approach and initiative, a larger-scale screening of the cloacal microbiota from reptiles in their natural habitats is necessary.

Herein, we isolated a variety of Gram-negative bacteria from the cloacal microbiota of five species of lizards (Sauria, Reptilia) and two species of tortoises (Testudines, Reptilia) as representatives of the herpetofauna in Bulgaria. The identified bacterial species are known to be opportunistic pathogens, both for their hosts and humans. In this study, we address the issue of the pathogenic potential of the isolated strains. The antibiotic susceptibility patterns to eight clinically important antimicrobials were determined. Biofilm formation and the extracellular production of enzymes considered as potential virulence factors were evaluated too. Several exoenzymes are part of the arsenal of extracellular or cell-associated virulence factors, along with toxins, adhesins, capsular polysaccharides, etc., produced by pathogens that are involved in the process of pathogenesis. A combination of enzymes (sialidases, proteases, hyaluronidases, lipases, gelatinases, etc.) contribute to the destruction of the integrity of mucous membranes and cellular structures, reductions in the viscosity of mucins, and the weakening of intercellular contacts [23]. These activities facilitate the penetration and spread of pathogens in the host. Very few studies on the microbiota of reptiles have addressed virulence factors such as enzymatic activity [24] or biofilm formation, and they have been limited to studying *Salmonella* in captive animals [25,26]. Therefore, the data provided are of concern for public health.

## 2. Materials and Methods

### 2.1. Sample Collection

A total of 86 lizards were sampled, namely the European green lizard (*L. viridis* Laurenti, 1768), n = 15; common wall lizard (*Podarcis muralis* Laurenti, 1768), n = 17; meadow lizard (*Darevskia praticola* Eversmann, 1834), n = 26; European snake-eyed lizard (*Ablepharus kitaibelii* Bibron and Bory de Saint-Vincent, 1833), n = 26; and European slow worm (*Anguis fragilis* Linnaeus, 1758); n = 2. Lizards were captured in an area located along the Dalbochitsa River valley, Ihtimanska Sredna Gora Mountain, northeast of the village of Gabrovitsa (42°15′12″ N 23°53′59″ E), 430–580 m above sea level. After sampling, they were released in their natural habitat.

A total of 24 adult tortoises were sampled: 12 spur-thighed tortoises (*Testudo graeca ibera* Pallas 1814) and 12 Hermann’s tortoises (*Testudo hermanni boettgeri* Mojsisovics 1889), with equal numbers of each gender. Tortoises were reared in a semi-free environment close to their natural habitat, at the Tortoise Rescue, Rehabilitation and Breeding Center in Banya village, Burgas District.

All sampled animals were clinically healthy.

Cloacal samples were collected using sterile cotton swabs inserted carefully into the cloaca pre-wiped with alcohol 70% and with a gentle rotating motion. The cotton swabs were placed immediately in Amies transport medium (Biolab Inc., Budapest, Hungary) and stored at 4 °C for 48 h until further processing in the laboratory.

The handling of all animals was conducted in accordance with the national legislation, respecting the policies concerning the ethical treatment of animals.

### 2.2. Isolation and Identification of Microorganisms

Cotton swabs were transferred to tubes with 5 mL Nutrient Broth (HIMedia Laboratories Pvt. Ltd., Maharashtra, India) to enrich the cultures and incubated at 37 °C for 24 h–48 h depending on bacterial growth. An initial screening of the mixed cultures was performed via inoculation in Petri dishes on the following selective and differentiation media: HiCrome UTI agar (HIMedia Laboratories Pvt. Ltd., Maharashtra, India), cetrimide agar (Merck Group, Darmstadt, Germany), TCBS agar (Biolab Inc., Budapest, Hungary), brilliant green agar (after pre-enrichment in Rappaport-Vassiliadis Broth) (Biolab Inc., Budapest, Hungary), and *Aeromonas* isolation medium (HIMedia Laboratories Pvt. Ltd., Maharashtra, India), followed by incubation for another 24 h. Isolated pure cultures obtained from single colonies were identified morphologically, microscopically (Gram staining), and biochemically using the identification kit MICROLATEST^®^ ID: ENTERO 24N Test (Cat.№ MLT00008, Erba Lachema, Brno, Czech Republic). Bacterial suspensions (1.0 McFarland) were inoculated in strips (0.1 mL in each well) and incubated at 37 °C for 24 h. Tests for catalase, glucose fermentation (OF test), cytochrome oxidase detection (OXI strip test), tryptophanase (INDOL test), and acetoin production (Voges–Proskauer reaction) were performed beforehand. Identification and interpretation of the obtained results were performed using the specialized software ErbaExpert Identification Program (www.erbalachema.com, accessed on 30 November 2022). The Automatic BD PhoenixTM M50 system (Becton, Dickinson and Company, Franklin Lakes, NJ, USA) was applied for a full biochemical characterization of the isolates according to the laboratory procedure, as described by the manufacturer. Briefly, the bacterial colonies (0.5 McFarland) were inoculated into the ID broth (Cat.№ 246001, ibid.) and 25 µL was transferred into the AST broth (Cat.№ 246003, ibid.) with one drop of an AST indicator solution (Cat.№ 246004, ibid.). The suspensions were poured in NMIC/ID-76 panels for Gram-negative bacteria (Cat.№ 448103, ibid.) and loaded into the instrument at 35 °C for 24 h. The obtained data were analyzed using EpiCentre™ software (V7.45A/V6.71A).

### 2.3. Antimicrobial Susceptibility Testing (AST)

The susceptibility of the isolated strains was tested using the Kirby–Bauer disc-diffusion method [27] against a panel of antibiotics: ampicillin (A, 10 µg/mL), amikacin (Am, 30 µg/mL), chloramphenicol (C, 30 µg/mL), ciprofloxacin (Cp, 5 µg/mL), cefazolin (Cfz, 30 µg/mL), gentamicin (G,10 µg/mL), tobramycin (Tb, 10 µg/mL), and tetracycline (T, 30 µg/mL) (BulBio, NCIPD Ltd., Sofia, Bulgaria). Bacterial cultures were incubated overnight in Nutrient Broth (HIMedia, India) at 37 °C. Bacterial suspensions standardized to McFarland 0.5 were plated on Mueller–Hinton agar (HIMedia Laboratories Pvt. Ltd., Maharashtra, India) Petri dishes and incubated at 37 °C for 24 h. Microorganisms were classified as susceptible (S), intermediate (I), or resistant (R) considering the interpretation criteria based on the inhibition zone diameters (mm) around each disc according to the CLSI and EUCAST guidelines [28,29]. The multiple antibiotic resistance (MAR) index was calculated using the following formula: MAR = a/b, where “a” represents the number of antibiotics the isolate is resistant to, and “b” represents the total number of antimicrobials tested [30].

### 2.4. Estimation of Enzymatic Activity

Proteolytic, lipolytic, and gelatinolytic enzyme production was evaluated via cultivation on calcium caseinate agar, Spirit Blue agar supplemented with 1% lipase reagent, and Nutrient agar supplemented with gelatin (8 g/L), respectively, with incubation at 37 °C for 24, 48 h, and 72 h. A positive reaction result was considered the appearance of a clear halo around the bacterial growth. For the visualization of gelatin hydrolysis, an ammonium sulfate solution was added on the surface [31]. As controls, *Aeromonas caviae* A40/02 (positive) was used for proteolytic production, and *Bacillus subtilis* (positive) and *Escherichia coli* ATCC 25922 (negative) were used for gelatinase production. Sialidase and sialate aldolase activities were determined using the colorimetric thiobarbiturate method [32]. Hyaluronidase production was assessed using the method of Patil and Chaudhari, 2017 [33].

### 2.5. Biofilm Formation

The biofilm-forming capacity of the isolated strains was evaluated via a semi-quantitative in vitro assay using 96-well U-bottomed polystyrene microtiter plates (Corning, NY, USA). Initially, bacterial cultures were grown overnight in Nutrient Broth (HIMedia, India) at 37 °C and used as a starting inoculum. Subsequently, bacterial suspensions with a concentration of 1 × 10^9^ cells/mL were diluted 1:100 in M63 minimal salt medium (0.02 M KH_2_PO_4_, 0.04 M K_2_HPO_4_, 0.02 M (NH_4_)_2_SO_4_, 0.1 mM MgSO_4_, and 0.04 M glucose, pH 7.5). Aliquots of 150 µL of the prepared suspensions were loaded into the wells of the plates in 6 replicates. The plates were sealed with parafilm to prevent desiccation and incubated at 37 °C for 24 h at static conditions. Biofilm forming was assessed using a semi-quantitative crystal violet assay. Non-adherent bacteria were discarded, and the wells were rinsed with Phosphate-buffered saline (PBS) before staining with 0.1% crystal violet followed by incubation at room temperature for 15 min. The unabsorbed dye was removed by rinsing the plates with PBS several times and the samples were solubilized with 70% ethanol. The absorbance of the solubilized samples was measured at 570 nm using a plate reader (INNO, Incheon, Republic of Korea). All experiments were conducted in duplicates, and the mean values with standard deviation (SD) were taken. Data analyses were carried out using Origin Pro 6.1. software (OriginLab Corporation, Northampton, MA, USA).

## 3. Results

From the cloacal samples of the lizards (n = 86) and tortoises (n = 24), a total of 253 and 138 isolates were obtained, respectively. The identified isolates belonged to 15 genera and 24 species (Table 1). The individual samples were found to be loaded with different numbers of bacterial species—we were able to identify from one to ten in each specimen. The most frequent isolates from the lizards were *Hafnia alvei* (from 39.5% of individuals), *Enterobacter amnigenus* biovar 2 (34.9%), and *Citrobacter braakii* (32.5%). In the vast majority of the tortoises (at least 75% of individuals), *Citrobacter braakii*, *Enterobacter cloacae*, *Klebsiella oxytoca/pneumoniae*, and *Salmonella enterica* subsp. *enterica* were encountered. According to some variations shown in their biochemical profiles, between one and six strains could be distinguished among the isolates of each bacterial species. A collection of 27 strains—one strain of each bacterial species to act as its representative—was selected for further experiments. The criteria for this selection was the confidence value being ≥97% for identification and the prevalence of this particular strain among the animal species studied.

### 3.1. Antibiotic Susceptibility

The newly isolated strains from the cloacal microbiota of the lizards and tortoises exhibited diverse resistance rates against the tested antibiotics (Table 2, section A). Susceptibility to amikacin, chloramphenicol, and ciprofloxacin was most widespread, with no resistance in any of the isolated strains detected. Four of the isolates (14.8%) demonstrated resistance to ampicillin (*E. amnigenus*, *K. oxytoca*^L^ *K. pneumoniae*, and *P. aeruginosa*) and to cefazolin (*H. alvei*, *E. vulneris*, *K. aerogenes*, and *P. aeruginosa*). Only two isolates (7.4%) showed multiresistance. The observed AMR (antimicrobial resistance) patterns were G/T in the *R. terrigena* strain and A/Cfz/T (MAR index 0.375) in the *P. aeruginosa* strain.

### 3.2. Extracellular Enzyme Production

**Figure 1:**
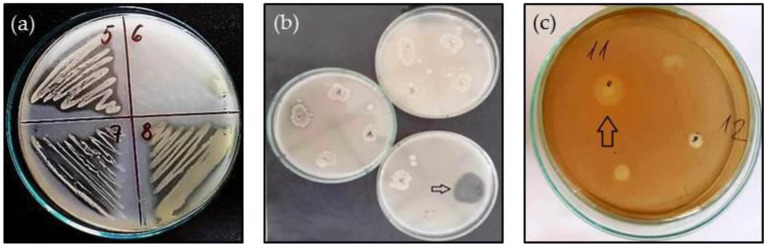
Examples of extracellular enzyme production: (**a**) protease (5—*R. aquatilis*^L^, 7—*S. plymuthica*, 8—*P. aeruginosa*); (**b**) gelatinase (*R. terrigena*, pointed with arrow); (**c**) hyaluronidase (11— *P. heimbachae*, pointed with arrow).

The isolates were tested for their extracellular production of enzymes considered as potential virulence factors—namely proteases, sialidases, gelatinases, lipases, and hyaluronidases, which may contribute to an infectious process. Most of the isolated strains showed extracellular production of at least one enzyme. The lack of expression of any exoenzyme was noted in only four isolates (Table 2, section B). The most widespread was proteolytic activity, detected in 77.8% (21/27) of the isolates. A positive reaction was recorded in *E. amnigenus*, *E. cloacae*^L,T^, *Citrobacter* spp., *P. aeruginosa*, *R. aquatilis*^L,T^, *E. coli*, *S. plymuthica*, *R. terrigena*, *Klebsiella* spp., *S. enterica*, *M. morganii*, and *P. agglomerans* (Figure 1a).

Extracellular sialidase activity was found in the culture supernatants of four isolates at 24 h of cultivation. The highest enzyme activity was measured in *C. freundii* (13.9 U/mL), followed in descending order by *S. plymuthica* (11.8 U/mL), *R. aquatilis*^T^ (10.93 U/mL), and *C. braakii*^L^ (7.25 U/mL). Sialidase production appeared to be a strain-specific feature, as it was recorded in *C. braakii*^L^ and *R. aquatilis*^T^ but not in *C.* braakii^T^ and *R. aquatilis*^L^, respectively. Sialate aldolase activity was not recorded in any of the isolates.

Lipase production was found only in *P. aeruginosa*. The halo around the bacterial growth was visible at 24 h of cultivation but more clearly formed after 48 h.

Three of the isolates, *P. aeruginosa*, *V. metschnikovii*, and *R. terrigena*, were positive for gelatinase production. The halo in *R. terrigena* appeared as early as 24 h of incubation, while the other two strains appeared later, at 72 h. Better visualization of the halo was achieved when saturated ammonium sulfate was added (Figure 1b).

Hyaluronidase activity was demonstrated only in *P. heimbachae* (Figure 1c).

Both protease and sialidase activity was exhibited by *C. braakii*^L^, *C. freundii*, *S. plymuthica*, and *R. aquatilis*^T^. *R. terrigena* and *P. aeruginosa* were positive for protease and gelatinase production. The latter was distinguished by the richest exoenzyme productivity of all isolates.

### 3.3. Biofilm-Forming Capacity

**Figure 2:**
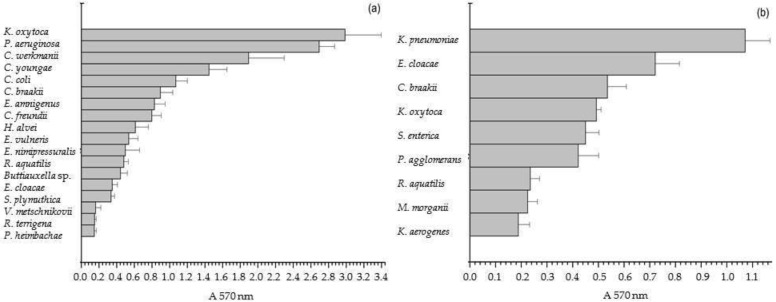
Biofilm-forming capacity of Gram-negative bacterial isolates from (**a**) lizards and (**b**) tortoises.

The analysis within the 24 h interval demonstrated a positive biofilm-forming ability across all examined isolates from the two reptile groups (Table 2, section C). They were categorized as weakly adherent (n = 6, 22.2%), moderately adherent (n = 11, 40.7%), and strongly adherent (n = 10, 37.3%). It is noteworthy that differences in the range of the biofilm-forming abilities of the representatives of the genera *Citrobacter* and *Klebsiella* were species- and strain-specific. Among the isolates from the lizards, *V. metschnikovii*, *P. heimbachae*, and *R. terrigena* were classified as weak biofilm producers (Figure 2a). Seven bacterial isolates, including *H. alvei*, *E. cloacae*^L^, *E. nimipressuralis*, *R. aquatilis*^L^, *Buttiauxella* sp., *E. vulneris*, and *S. plymuthica*, were categorized as moderate biofilm producers, with values ranging from 0.336 ± 0.038 to 0.611 ± 0.10. A significant biofilm-forming ability was observed in 29.6% (8/27) of the isolates, including *E. amnigenus*, *C. braakii*^L^, *C. youngae*, *C. freundii*, *C. werkmanii*, *P. aeruginosa*, *K. oxytoca*^L^, and *E. coli.* Two isolates, *P. aeruginosa* 2.694 ± 0.097 and *K. oxytoca*^L^ 2.987 ± 0.120, were distinguished by their excellent biofilm-forming capacity.

Among the isolates from the tortoises, a low biofilm-forming capacity was shown by *K. aerogenes*, *R. aquatilis*^T^, and *M. morganii*, ranging from 0.188 ± 0.044 to 0.234 ± 0.036 and 0.224 ± 0.037, respectively. Four strains were identified as intermediate biofilm producers, including *C. braakii*^T^, *K. oxytoca*^T^, *S. enterica*, and *P. agglomerans*, with values ranging from 0.421 to 0.535, while *E. cloacae*^T^ and *K. pneumoniae* could be determined as strong with values of 0.721 ± 0.094 and 1.071 ± 0.092, respectively (Figure 2b).

**Table 2:** Antibiotic susceptibility, extracellular enzyme production, and biofilm-forming capacity of the isolates from the reptile cloacal microbiota.

Bacterial Isolates	A. Antibiotics								B. Extracellular Enzyme Production					C. Biofilm-
	A	Am	C	Cp	Cfz	G	Tb	T	Sial	Prot	Lip	Gel	Hyal	
**from lizards**														
*H. alvei*	S	S	S	S	R	S	S	S	−	−	−	−	−	++
*E. amnigenus*	R	S	S	S	S	S	S	S	−	+	−	−	−	+++
*E. cloacae* ^L^	S	S	S	S	S	S	S	S	−	+	−	−	−	++
*E. nimipressuralis*	S	S	S	S	S	S	S	S	−	−	−	−	−	++
*C. braakii* ^L^	S	S	S	S	S	S	S	S	+	+	−	−	−	+++
*C. youngae*	I	S	S	S	I	S	S	S	−	+	−	−	−	+++
*C. freundii*	S	S	S	S	S	S	S	S	+	+	−	−	−	+++
*C. werkmanii*	S	S	S	S	I	S	S	S	−	+	−	−	−	+++
*P. aeruginosa*	R	S	S	S	R	S	S	R	−	+	+	+	−	++++
*R. aquatilis* ^L^	I	S	S	S	I	S	S	S	−	+	−	−	−	++
*K. oxytoca* ^L^	R	S	S	S	S	S	S	S	−	+	−	−	−	++++
*Buttiauxella* sp.	S	S	S	S	S	S	S	S	−	−	−	−	−	++
*V. metschnikovii*	S	S	S	S	S	S	S	S	−	−	−	+	−	+
*E. vulneris*	S	S	S	S	R	S	S	S	−	−	−	−	−	++
*E. coli*	S	S	S	S	S	S	S	S	−	+	−	−	−	+++
*S. plymuthica*	S	S	S	S	I	S	S	S	+	+	−	−	−	++
*P. heimbachae*	S	S	S	S	S	S	S	S	−	−	−	−	+	+
*R. terrigena*	S	S	S	S	S	R	R	S	−	+	−	+	−	+
**from tortoises**														
*C. braakii* ^L^	S	S	S	S	I	S	S	nd	−	+	−	−	−	++
*E. cloacae* ^L^	S	S	S	S	I	S	S	nd	−	+	−	−	−	+++
*K. oxytoca* ^L^	S	S	S	S	S	S	S	nd	−	+	−	−	−	++
*K. pneumoniae*	R	S	S	S	S	S	S	nd	−	+	−	−	−	+++
*K. aerogenes*	S	S	S	S	R	S	S	nd	−	+	−	−	−	+
*S. enterica*	S	S	S	S	S	S	S	nd	−	+	−	−	−	++
*R. aquatilis* ^L^	I	S	S	S	S	S	S	nd	+	+	−	−	−	+
*M. morganii*	S	S	I	S	I	S	S	nd	−	+	−	−	−	+
*P. agglomerans*	S	S	S	S	S	S	S	nd	−	+	−	−	−	++

Legend: Section A: susceptible (S), intermediate (I), resistant (R); ampicillin (A), amikacin (Am), chloramphenicol (C), ciprofloxacin (Cp), cefazolin (Cfz), gentamicin (G), tobramycin (Tb), tetracycline (T), nd—not determined; Section B: sialidase (Sial), protease (Prot), lipase (Lip), gelatinase (Gel), hyaluronidase (Hyal); Section C: biofilm-forming capacity: weak (+); moderate (++); strong (+++); excellent (++++).

## 4. Discussion

A large proportion of the Gram-negative bacteria we identified have been described as opportunistic pathogens in animals and humans [5,14]. We found a high prevalence of *Citrobacter* spp., *Enterobacter* spp., *Klebsiella* spp., and *P. aeruginosa* in the cloacal swabs. These genera are also common in the cloacal microflora of various reptile species. [7,11,16,34].

Wildlife is not expected to play a fundamental role in the emergence of antibiotic resistance, as it is primarily associated with anthropogenically affected habitats through selective pressure. However, wild animals, including reptiles, may harbor antibiotic-resistant pathogens. Once resistant bacteria are acquired by wild animal hosts, they can act as their reservoir, transmitting them through horizontal transfer [35]. The lower rates of drug resistance in bacteria isolated from the fecal microbiota of free-living reptiles may be due to the lesser loads of antibiotics in their environment compared to surroundings where antibiotic use is common [34]. Antimicrobial sensitivity testing applied to clinical isolates from captive reptiles is of great importance to define an appropriate treatment to avoid the development of resistant bacterial isolates [5], but its performance in wild-type strains is severely limited [34,36].

Our findings revealed that most of the isolates were susceptible to the conventional antibiotics tested, but also that some of the opportunistic Gram-negative bacteria circulating in the studied reptile populations showed multiple resistance. Organisms with MAR indices greater than ≥0.2 suggest the presence of plasmids containing one or more resistance genes, each encoding an antibiotic resistance phenotype [37]. The isolated *P. aeruginosa* strain manifested resistance to the largest number of antibiotics, including tetracycline, and showed less susceptibility to chloramphenicol (smaller inhibition zone) compared to the other isolates. *Pseudomonas aeruginosa* often shows a high propensity for multidrug resistance (MDR) when the strain is not susceptible to one or more antibiotics in at least three or more antibiotic classes [7,38]. Responsible for this ability are features of the outer cell membrane, acting as a barrier; efficient efflux transport systems; genes that encode enzymes; inactivating drugs; and R-plasmids [38,39]. *Pseudomonas* spp. are frequently present in the oral and cloacal microbiota of different reptile species, both in clinically healthy and symptomatic individuals, causing dermatitis, stomatitis, cloacitis, abscesses, ear and respiratory infections, and septicemia, with consequent death [5,14]. *Raoultella terrigena*, which only showed resistance to two aminoglycoside antibiotics, is a rare opportunistic pathogen, but this infection and MDR profile has a high mortality rate [40].

The non-susceptibility to beta-lactams (ampicillin and cefazolin) demonstrated by several of the isolates may suggest natural mechanisms of antibiotic resistance, such as constitutive chromosomal AmpC beta-lactamases and cephalosporinases [36]. MDR has been reported in strains of *P. aeruginosa*, *C. freundii*, *M. morganii*, *K. pneumoniae*, *E. coli*, *E. amnigenus*, and *H. alvei* obtained from various reptiles, both in the wild and in captivity [34,36,38,41]. *Salmonella* is of particular interest because this pathogen is considered an important zoonotic agent of public concern. Reptiles are very common asymptomatic carriers of a wide variety of serovars [19,42,43]. RAS (reptile-associated salmonellosis) can lead to serious infections, especially in at-risk groups, such as children under 5 years old, pregnant women, or immunocompromised patients, which could result in septicemia, miscarriage, or death [19]. However, none of the samples from the studied lizards were positive for *Salmonella*. Although it is one of the pathogens most commonly present in cloacal samples, *Salmonella*-free wild populations of various reptile species are not unprecedented [15,18]. Moreover, Schmidt et al. (2014) found a *Salmonella* burden only in *Vipera berus*, inhabiting the same area as *Anguis fragilis* and *Natrix natrix*, where this pathogen was not detected [16]. We found a high prevalence of *S. enterica* subsp. *enterica* in the studied tortoise population—75% of individuals (18/24–11 *T. graeca* and 7 *T. hermanni*) were carriers. This rate of positive samples corresponds with the findings of Casalino et al., 2021 [44], but is higher than that reported by Marenzoni et al., 2022 [2]. The isolated strain of *S. enterica* subsp. *enterica* showed susceptibility to all of the tested antibiotics, analogous to the results obtained by other authors [2]. Reptile-associated *Salmonella* shows less resistance than that from farm animals [22]. Resistance to streptomycin has been reported in *Salmonella* originating from free-living snakes, probably due to aminoglycoside-modifying genes in the snake isolates [16,22] and to penicillin in the isolates from tortoises [38]. However, pet reptiles could be a potential source of multidrug-resistant *Salmonella* [42,43]. The increase in multidrug-resistant *Salmonella* strains enhances the risk of therapeutic failure in cases of life-threatening salmonellosis in human and veterinary medicine [43].

During an infectious process, pathogens produce a wide range of extracellular enzymes known as virulence factors, which directly or indirectly participate in the colonization of the organism by facilitating adhesion to surfaces, breaking down the host’s physical barriers (cell walls and intercellular connections), modulating the immune response, etc. [23,45]. An important role in the mechanisms of pathogenesis, along with other extracellular hydrolytic enzymes, is played by bacterial proteases. They promote the penetration and efficient dissemination of pathogens within a host by participating in the destruction of cellular structures and reducing the integrity between tissues [46,47]. Moreover, proteolytic enzymes are involved in the inactivation of components of the host’s immune defense (e.g., immunoglobulin, IgA) [48]. Most of the strains we managed to isolate turned out to be protease producers. Several of them produced sialidases as well. Sialidases cleave terminal sialic residues from a number of the cell surface structures, such as glycoproteins, glycolipids, oligosaccharides, etc. [49]. The release of sialic acid uncovers the receptors to which pathogens or toxins bind, thus deepening the process of pathogenesis. Furthermore, removing the first protective barrier, sialidases “pave the way” for the remaining hydrolytic enzymes, which contributes to the degradation of cellular components and disruption of the integrity of the tissues. In most commensals, the released sialic acid is degraded to pyruvate and N-acetylmannosamine by the enzyme sialate aldolase and absorbed as a carbon and energy source [49]. Since sialate aldolase activity was not recorded in any of the isolates, we suggest that in the sialidase-positive isolates, the sialic acid was not cleaved to be digested but rather to allow other glycosidases access to the basic sugars of the sialoglycans. As far as we know, the available literature lacks data on the production of sialidases in members of the genera *Serratia*, *Citrobacter*, and *Rahnella*, which we found to be sialidase-positive.

Bacterial lipolytic activity is involved in nutrient acquisition through the degradation of membrane lipids, and thus may cause harm to the host [50]. Bacterial lipases are considered a virulence factor because they hydrolyze triglycerides, thereby disrupting the lipid components of cell membranes. The pathogenic effect of lipolytic enzymes is expressed not only in their direct hydrolytic action, but also in indirectly influencing various cellular signaling pathways, as well as in the modulation of the immune response [51]. Lipase production has been reported in *K. pneumoniae*, *K. oxytoca*, *P. agglomerans*, and *Salmonella* strains from the cloacal microbiota of amphibians and reptiles [24,25]. Among the isolates from our study, lipase production was restricted to *P. aeruginosa* only. The mechanism of the secretion of this enzyme has been studied in detail in *P. aeruginosa* and *P. fluorescens* [52].

Gelatinase is a protease capable of hydrolyzing gelatin, collagen, hemoglobin, and other small amounts of biologically active peptides [50]. Its production was demonstrated in three isolates, including *P. aeruginosa*. The newly isolated *P. aeruginosa* strain was distinguished as having the richest exoenzyme arsenal compared to all of the other isolates.

Only *P. heimbachae* manifested a positive hyaluronidase reaction. This enzyme breaks down hyaluronic acid, which is a component of the extracellular matrix of a number of tissues (connective, epithelial, and nervous) and organs (joints, skin, heart valves, vitreous body of the eye, etc.), thereby facilitating the destruction of intercellular connections and the penetration of pathogens in the host [53].

Biofilm formation is regarded as a key mechanism of virulence in Gram-negative pathogenic bacteria, especially in those responsible for chronic infections, aiding bacterial survival and persistence within the host [54]. Bacteria with the capacity to form biofilms can be more resistant to antimicrobials than planktonic cells. Bacterial biofilms provide an adequate environment for the interaction between microorganisms and the acquisition of resistance genes, enabling the maintenance of bacteria that could act as reservoirs for these genes [55]. We are not aware of any data in the available literature regarding biofilm-forming abilities in wild-type strains from the reptile cloacal microbiota, but our study revealed that all isolates showed such a capability to some extent. *P. aeruginosa*, considered a strongly adherent strain, was also multiresistant. In contrast, the low biofilm-forming capacity of *V. metschnikovii* and *P. heimbachae* was complemented by their high antibiotic susceptibility to all applied antibiotics.

## 5. Conclusions

Our study is a first report concerning the epidemiological status of reptile species found in Bulgaria. A variety of Gram-negative bacteria, which are also alleged agents of infection in humans, were found in the cloacal microbiota of free-living lizards and tortoises. Manifestations of resistance to two or more antibiotics, the expression of different virulence factors, and biofilm-forming capacity are indicators of the pathogenic potential of some of the isolated strains, which should not be ignored. The isolated *P. aeruginosa* strain apparently had the highest pathogenicity, proven by its multidrug resistance, rich extracellular enzyme production, and strong biofilm-forming ability. Due to the opportunistic nature of Gram-negative bacteria residing in the cloaca of cold-blooded animals, it is recommended that people who are often in close contact with various reptiles—e.g., herpetologists, zookeepers, pet owners, veterinarians, etc.—be cautious about the possible transmission of infections. The monitoring of reptiles, both captive and free-range, could be of great importance in elucidating the spread of zoonotic bacteria and their epidemiological significance.

## Figures and Tables

**Table 1:** List of Gram-negative bacteria isolated from the reptile cloacal microbiota.

Bacterial Species	Total Number of Positive Individuals/ Percentage	Hosts/Number of Positive Individuals			Number of Strains
	**Lizards n = 86**				
*Hafnia alvei*	34 (39.5%)	*L. viridis/*5 *A. kitaibelii/*15	*P. muralis/*5 *A. fragilis/*1	*D. praticola/*8	5
*Enterobacter amnigenus biovar 2*	30 (34.9%)	*L. viridis/*1 *A. kitaibelii/*10	*P. muralis/*5 *A. fragilis/*1	*D. praticola/*13	4
*Enterobacter cloacae* ^L^	11 (12.8%)	*P. muralis/*7	*D. praticola/*4		2
*Enterobacter nimipressuralis*	2 (2.3%)	*P. muralis/*2			1
*Citrobacter braakii* ^L^	28 (32.5%)	*L. viridis/*4 *A. kitaibelii/*3	*P. muralis/*9 *A. fragilis/*1	*D. praticola/*11	4
*Citrobacter youngae*	16 (18.6%)	*L. viridis/*3 *A. kitaibelii/*1	*P. muralis/7* *A. fragilis/*1	*D. praticola/*4	2
*Citrobacter freundii*	10 (11.6%)	*L. viridis*/2 *A. kitaibelii/*4	*P. muralis/*1	*D. praticola/*3	3
*Citrobacter werkmanii*	1 (1.2%)	*L. viridis*/1			1
*Pseudomonas aeruginosa*	24 (27.9%)	*L. viridis/*1 *A. kitaibeli/*4	*P. muralis/*8 *A. fragilis/*2	*D. praticola/*9	5
*Rahnella aquatilis* ^L^	22 (25.6%)	*L. viridis/*2 *A. fragilis/*1	*P. muralis/*4	*D.praticola/*15	6
*Klebsiella oxytoca* ^L^	18 (20.9%)	*L. viridis/*6 *A. kitaibelii/*3	*P. muralis/*2 *A. fragilis/*1	*D. praticola/*6	5
*Buttiauxella* sp.	16 (18.6%)	*P. muralis/*4	*D.praticola/*11	*A. kitaibelii/*1	2
*Vibrio metschnikovii*	13 (15.1%)	*D. praticola/*7	*A. kitaibelii/*6		1
*Escherichia vulneris*	10 (11.6%)	*L. viridis/*2	*D. praticola/*5	*A. kitaibelii/*3	1
*Escherichia coli*	4 (4.6%)	*L. viridis/*2	*P. muralis/*2		2
*Serratia plymuthica*	9 (10.5%)	*L. viridis/*1	*P. muralis/*3	*A. kitaibelii/*5	2
*Providencia heimbachae*	4 (4.6%)	*L. viridis/*2	*D. praticola/*2		1
*Raoultella terrigena*	1 (1.2%)	*L. viridis/*1			1
Total number of isolates	253			Total number of strains	48
	**Tortoises n = 24**				
*Citrobacter braakii* ^T^	21 (87.5%)	*T. graeca/*10	*T. hermanni/*11		3
*Enterobacter cloacae* ^T^	19 (79.2%)	*T. graeca/*8	*T. hermanni/*11		2
*Klebsiella oxytoca* ^T^	19 (79.2%)	*T. graeca/*10	*T. hermanni/*9		3
*Klebsiella pneumoniae*	19 (79.2%)	*T. graeca/*10	*T. hermanni/*9		3
*Klebsiella aerogenes*	13 (54.2%)	*T. graeca/*8	*T. hermanni/*5		3
*Salmonella enterica* subsp. *enterica*	18 (75%)	*T. graeca/*11	*T. hermanni/*7		2
*Rahnella aquatilis* ^T^	17 (70.1%)	*T. graeca/*9	*T. hermanni/*8		2
*Morganella morganii*	7 (29.2%)	*T. graeca/*2	*T. hermanni/*6		1
*Pantoaea agglomerans*	5 (20.1%)	*T. graeca/*4	*T. hermanni/*1		1
Total number of isolates	138			Total number of strains	20

The ^L^ and ^T^ indices distinguish isolates from lizards and tortoises in the bacterial species common to both groups of reptiles.

## Data Availability

Data are contained within the article.
